# A case report of exogenous lipoid pneumonia associated with avocado/soybean unsaponifiables

**DOI:** 10.1186/s12890-019-0997-1

**Published:** 2019-12-03

**Authors:** Jacques BOUTROS, Marine MUZZONE, Jonathan BENZAQUEN, Michael LEVRAUT, Charles-Hugo MARQUETTE, Fanny ROCHER, Yann DIASCORN, Bernard PADOVANI, Véronique HOFMAN, Sylvie LEROY

**Affiliations:** 1 Department of Pulmonary Medicine, Université Côte d’Azur, CHU de Nice, FHU OncoAge, 30 avenue de la voie Romaine, CS51069, 06001 Nice, France; 2 Université Côte d’Azur, CNRS UMR7284, Inserm U1081, Institute of Research on Cancer and Ageing (IRCAN), Nice, France; 3 Université Côte d’Azur, CHU de Nice, Regional Pharmacovigilance Center, Nice, France; 4 Department of Radiology, Université Côte d’Azur, CHU de Nice, Nice, France; 5 Laboratory of Clinical and Experimental Pathology, Université Côte d’Azur, CHU de Nice, FHU OncoAge, Nice, France; 6 Université Côte d’Azur, CNRS UMR 7275 - Institut de Pharmacologie Moléculaire et Cellulaire, Sophia Antipolis, France

**Keywords:** Exogenous lipoid pneumonia, Avocado oil, Soybean oil, Hiatal hernia, Gastroesophageal reflux disease

## Abstract

**Background:**

Exogenous lipoid pneumonia is a rare disease resulting from intra-alveolar accumulation of lipids of mineral, vegetal, or animal origin, that induce a foreign body type of inflammatory reaction in the lungs. Gastroesophageal reflux disease and other esophageal abnormalities have often been associated with this disease.

**Case presentation:**

We herein report the case of an 83-year-old patient in whom a follow-up chest computed tomography scan, for a lingular consolidation, showed multifocal ground glass and consolidative opacities with areas of low attenuation, suggestive of exogenous lipid pneumonia. The patient had been on piascledine capsules (avocado/soybean unsaponifiables) for 20 years and had a hiatal hernia with documented gastroesophageal reflux disease. After thorough history taking, no other predisposing factors were found. The diagnosis was confirmed using oil red staining of bronchoalveolar lavage showing lipid-laden macrophages and extracellular lipid droplets.

**Conclusions:**

To our knowledge, this is the first case of ELP secondary to avocado/soybean unsaponifiables in the literature.

## Background

Exogenous lipoid pneumonia (ELP) is a rare lung condition due to the inflammatory reaction generated by the presence of foreign fatty substances in the alveoli. ELP can be suspected when a clinical history of inhalation or aspiration of fatty substances and chest imaging are compatible [1–3]. Confirmation of the diagnosis requires special staining of bronchoalveolar lavage (BAL) fluid or lung biopsies [4, 5]. We present an unusual case of ELP caused by avocado/soy unsaponifiables (ASU).

## Case presentation

**Fig. 1:**
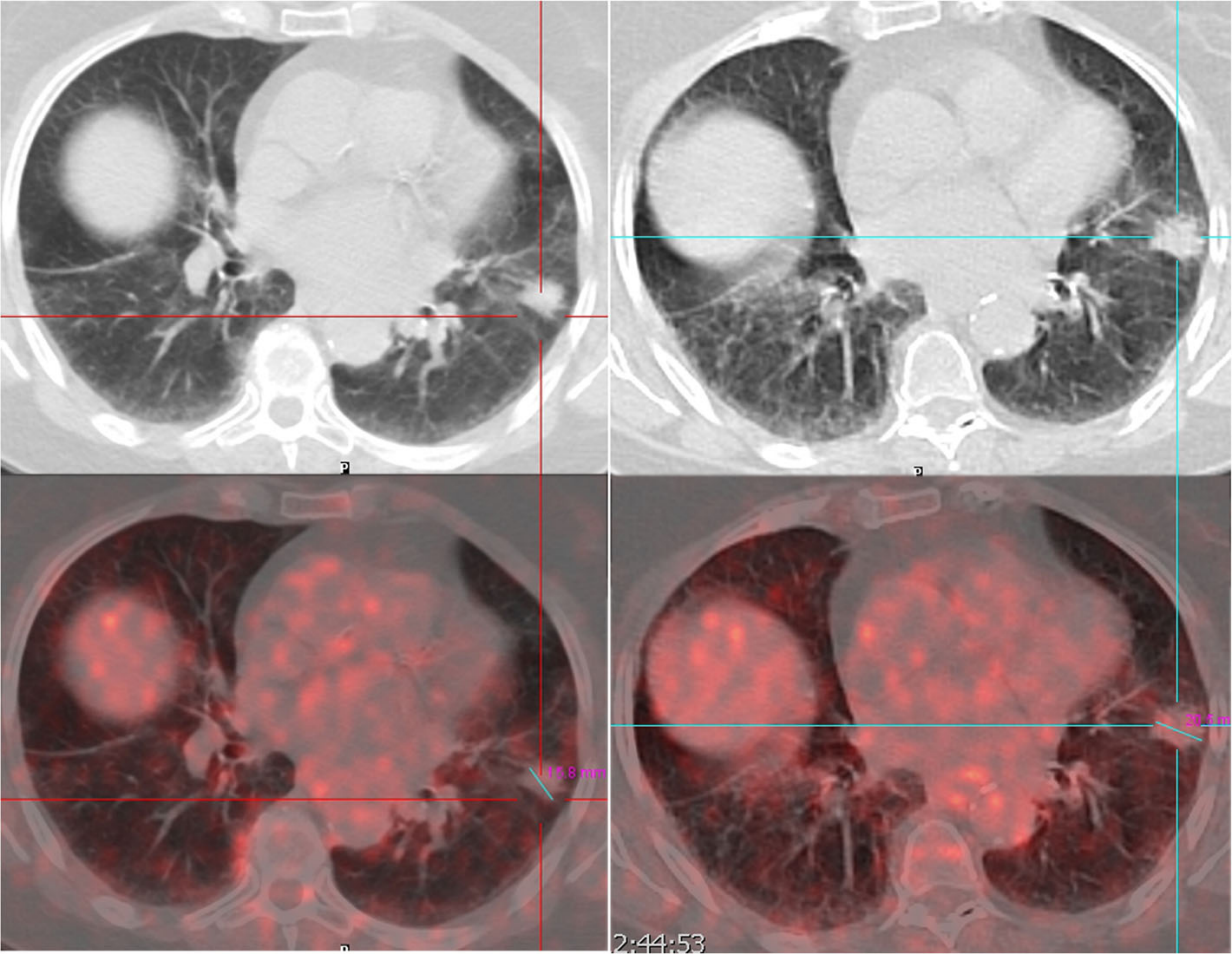
Left: baseline PET scanner, 15.6 mm lingular consolidation, no abnormal FDG uptake; right: follow up PET scanner, 15 months later, 20.5 mm lingular consolidation, no abnormal FDG uptake

An 83-year old never-smoking female consulted in 2017 for a dry nocturnal cough, without dyspnea**.** An incidental lingular consolidation had been detected on a chest computed tomography (CT) scan in 2010, and was followed by regular chest CTs and positron emission tomography– computed tomography (PET/CT) scans until 2016. She had had two undiagnostic bronchoscopies and refused further invasive examinations including thoracoscopy. She had a medical history of osteoarthritis and hiatal hernia. Until her visit in 2017, she was in good general condition, had no signs of respiratory disease, and her physical examination was normal. The PET/CT scan (Fig. 1) performed in 2015 showed no abnormal metabolic activity. The follow-up PET-CT (Fig. 1) performed 15 months later showed a slight increase in size of the consolidation (20 mm) without abnormal uptake.

**Fig. 2:**
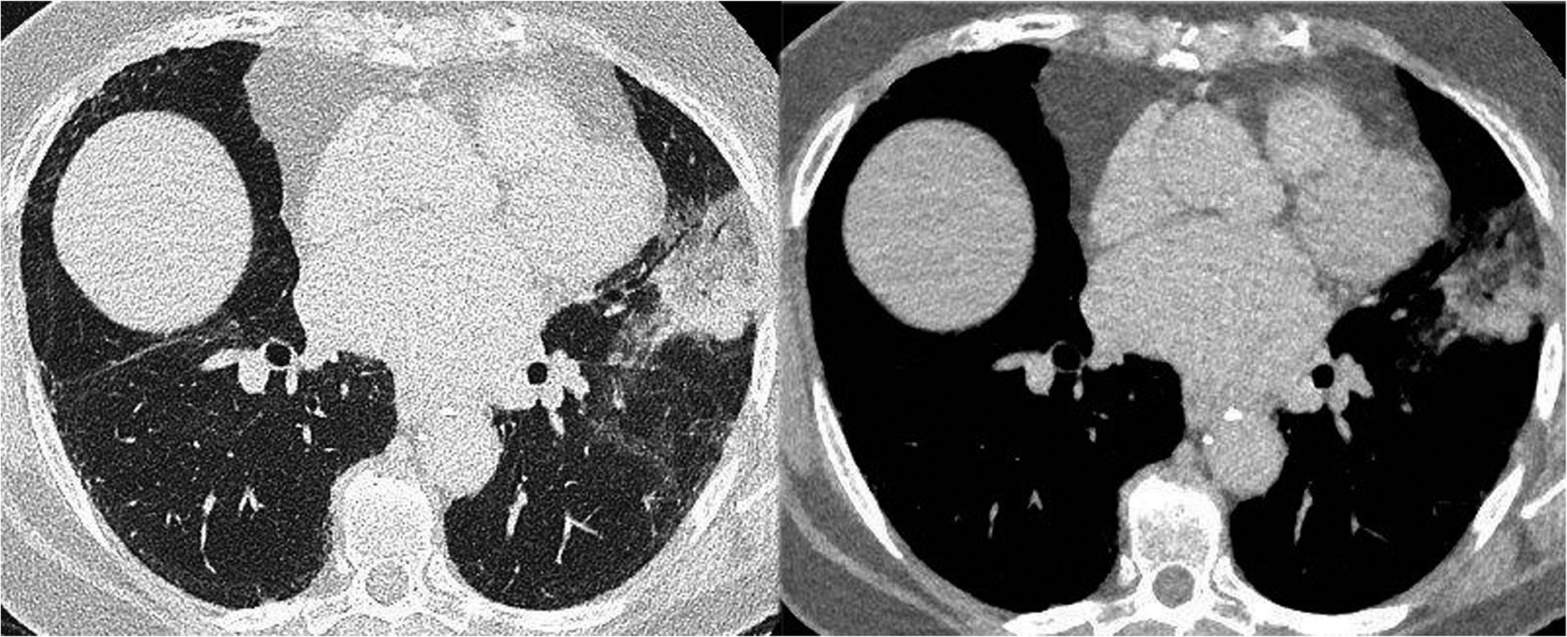
Follow-up TCT scan in 2018 showing lingular consolidation with multifocal ground-glass opacities in the lingular and culminal segments (left) and areas of fat attenuation (right)

**Fig. 3:**
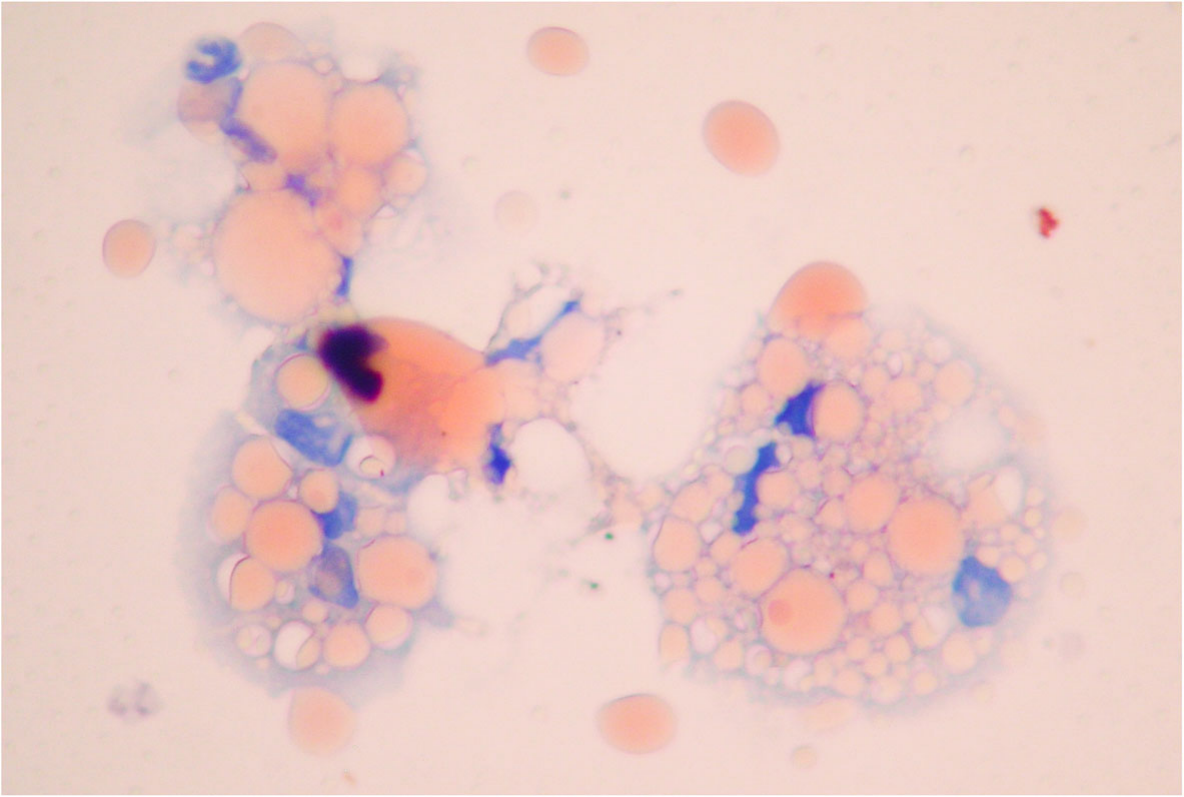
Alveolar macrophages in bronchoalveolar lavage with intracytoplasmic micro and macrovacuoles filled with lipid associated with extracellular lipid droplets (oil red, original magnification × 1000)

When she presented in 2017, the chest examination was still normal. Pulmonary function tests (PFTs) showed the onset of a restrictive disease with 3.69 Liters (75% of predicted) of total lung capacity versus 5.19 Liters 1 year earlier, without weight gain. No oxygen desaturation was detected at rest or during a 6-min walking test. This time, a CT scan showed multifocal ground-glass and consolidative opacities with heterogeneous densities, including areas of low attenuation, indicative of intrapulmonary lipid, in the left upper lobe (LUL) (Fig. 2). After thorough history taking concerning exposure to fatty substances, the patient denied any form of inhalation of volatile hydrocarbons. However, she had been on ASU twice daily for 20 years to treat osteoarthritis. Gastroesophageal reflux disease (GERD) was documented using 24-h pH-metry showing 90 acid-reflux episodes per 24 h, despite treatment with esomeprazole. Bronchoscopy was performed, and the BAL fluid was macroscopically turbid white. Oil red O staining of BAL showed lipid-laden macrophages (Fig. 3) and extracellular lipid droplets. Cultures of the BAL were negative.

After confirmation of the diagnosis, in the absence of other fatty-substance exposure, a presumptive diagnosis of ELP caused by ASU aspiration in a patient with GERD was established. This treatment was stopped. No surgical treatment of her hiatal hernia was suggested in this patient who had refused invasive procedures. One year later, the patient was clinically stable, her cough regressed and her PFTs normalized. Her chest CT showed persistent lingular consolidation and regression of the ground-glass opacities.

## Discussion and conclusions

ELP is an unusual form of pneumonitis, caused by exogenous lipids of mineral, vegetal, or animal origin [6], reaching the alveoli by inhalation of volatile hydrocarbons [7–9], or by aspiration of oil-based substances. Most cases of ELP result from mineral oil aspiration found in laxatives and oily nose drops [1].

The risk of ELP is increased in multiple esophageal disorders and GERD [1, 4, 10]. Hiatal hernia has been associated with ELP in 2 cases reported in the literature [11, 12].

Diagnosing ELP is based on a history of exposure to exogenous fat, compatible radiological findings, and a demonstration of lipids in lung biopsy specimens or BAL [1, 6, 10, 13, 14]. In a French case series, GERD was associated with ELP in 50% of cases [1]. Lipids tend to overlay gastrointestinal fluids in static situations, making patients more vulnerable to chronic aspiration of fat, especially in hiatal hernia with GERD [12]. Our patient had typical ELP with a compatible CT scan and BAL. Although it has never been described in the literature, the most probable culprit in this context was the 300 mg non-gastro-resistant capsules of ASU capsules [15–17]. We consulted the international databases of “Expanscience Laboratoires”, the manufacturer of the drug, looking for similar adverse effect in the drug safety databases, but we did not find any. Although the patient’s cough may arguably have been due to the ELP itself, its nocturnal timing and the GERD-compatible 24-h pH-metry suggested symptomatic nocturnal GERD.

ELP is often insidious, and in our patient, the initial lingular opacity could be an inaugural sign of the disease [2], even though no low-attenuation areas were seen within it. Both PET scans did not show any abnormal uptake. We currently do not know the value of PET scans in ELP evaluation. Nonetheless, several papers have reported cases of ELP mimicking neoplastic nodules on PET-CT, with moderate uptake of up to 4.4 of SUVmax [18–20].

Although ELP is uncommon, the pneumologist should have a high degree of suspicion whenever a history and imaging results are suggestive of the disease. Thorough search of the culprit source is essential to reverse the disease. Our case is the first to report ELP caused by ASU, readily commercialized as dietary joint supplements.

**Case Report Timeline**

**Table:** 

2010	April 2015	June 2016	December 2017–March 2018	March 2019
**Chest CT**: lingular consolidation	**Follow-up PET/CT scan:** non-hypermeta-bolic lingular consolidation **Pulmonary function tests:** normal	**Follow-up PET/CT scan:** persistant non-hypermeta-bolic lingular consolidation	**Clinically:** dyspnea and cough **Chest CT:** lingular consolidation compatible with lipoid pneumonia **Bronchoscopy with broncho-alveolar lavage:** compatible with lipoid pneumonia **Pulmonary function test:** restrictive pattern. **pH-metry:** compatible with GERD **Decision:** stop treatment with avocado/soybean unsaponifiables	**Clinically:** improvement **Chest CT:** regression of ground-glass opacities. Persistence of lingular consolidation **Pulmonary function test:** normal

## Data Availability

The datasets used and/or analyzed during the current study are available from the corresponding author on reasonable request.

## Abbreviations

ASU: Avocado/soy unsaponifiable
BAL: Bronchoalveolar lavage
CT: Computed tomography
ELP: Exogenous lipoid pneumonia
GERD: Gastroesophageal reflux disease
LUL: Left upper lobe
PET/CT: Positron emission tomography – computed tomography
PFTs: Pulmonary function tests
